# Neurophysiological and biological effects of music-based interventions in ADHD: a mini review

**DOI:** 10.3389/fpsyt.2026.1901818

**Published:** 2026-07-31

**Authors:** Michaela M. Zrelski, Bettina L. Serrallach, Christine Groß

**Affiliations:** 1 Department of Child and Adolescent Psychiatry, Saarland University, Homburg, Germany; 2 Institute of Diagnostic and Interventional Neuroradiology, Inselspital, University Hospital Bern, University of Bern, Bern, Switzerland; 3 SRH University, School of Health, Education and Social Sciences, Heidelberg, Germany; 4 Jāzeps Vītols Latvian Academy of Music, Riga, Latvia

**Keywords:** ADHD, auditory processing, biomarkers, cortisol, EEG, MEG, music therapy, music-based interventions

## Abstract

Attention-deficit/hyperactivity disorder (ADHD) is increasingly recognized as a lifespan neurodevelopmental condition. Because musical perception and production engage auditory, attentional, motor, emotional, and regulatory systems simultaneously, music-based interventions have emerged as promising complementary approaches for ADHD. However, their underlying neurophysiological and systemic biological correlates remain insufficiently understood. This mini review synthesizes evidence on behavioral, neurophysiological, and biological effects of music-based interventions in ADHD using a narrative approach. Current evidence suggests that music-based interventions may influence domains related to attention, impulse control, emotional regulation, and broader behavioral functioning. Neurophysiological findings indicate potential effects on auditory processing, interhemispheric synchronization, and cortical oscillatory activity, as demonstrated using MRI, MEG, and EEG. Preliminary systemic biological findings further suggest modulation of stress-related and autonomic processes, including cortisol, serotonin, heart rate, blood pressure, and heart-rate variability. Importantly, almost all studies identified in this review were conducted in children and adolescents, despite ADHD being increasingly recognized as a lifespan condition. Consequently, the generalizability of current findings to adults with ADHD remains uncertain. Furthermore, the available evidence base is limited by small sample sizes, inconsistent reporting standards, multimodal intervention designs, and insufficient control of medication effects. Overall, music-based interventions represent a promising but still insufficiently characterized approach for ADHD. Future research should integrate behavioral, neurophysiological, and biological outcomes within adequately powered, mechanistically informed, and developmentally sensitive study designs to clarify therapeutic mechanisms and clinical relevance across the lifespan.

## 1 Introduction

Attention-deficit/hyperactivity disorder (ADHD) is among the most common neurodevelopmental disorders, with a worldwide pooled prevalence of ~5% in children and adolescents and ~2.5% in adults (1, 2). ADHD is increasingly recognized as a lifespan condition (3). It is characterized by developmentally inappropriate levels of hyperactivity, impulsivity, and/or inattention. Beyond its core behavioral features, ADHD has been associated with alterations in executive functioning, and a wide range of timing-related processes, including deficits across motor and perceptual timing as well as temporal foresight (4–6). Moreover, ADHD is linked to neurochemical dysregulation, particularly in dopaminergic and noradrenergic systems (7–9), but also in other neurotransmitter systems, including serotonin (9, 10). These perspectives are relevant to music, as musical perception and production rely strongly on temporal precision, rhythm processing, sensorimotor integration, and interaction of auditory and attentional systems, while music may additionally modulate dopaminergic pathways (11, 12). The auditory cortex is broadly connected with subcortical, prefrontal, and parietal brain regions involved in attentional regulation, and ADHD has repeatedly been reported to overlap with auditory processing difficulties (13–16).

Current treatment approaches for ADHD remain centered on medication and behavioral interventions. Although these treatments are effective for many patients, they are associated with important limitations, including side effects, variable responses, and an incomplete understanding of the neurobiological heterogeneity underlying clinical presentations (17, 18). In this context, music-based approaches have emerged as promising complementary candidates. Music is widely accessible, emotionally engaging, and capable of simultaneously recruiting auditory, temporal, motor, attentional, emotional, and regulatory processes through the engagement of distributed neural and neurochemical systems (19–21).

Despite growing interest, the current evidence base remains limited. Our structured literature search yielded a small number of studies, highlighting a substantial scientific underrepresentation of music-based interventions in ADHD research. Moreover, while recent reviews such as Saville et al. (22) have summarized the effects of music-based interventions in ADHD across the lifespan, existing data predominantly focus on behavioral and cognitive aspects. Biological processes and potential connections between behavioral outcomes and underlying mechanisms remain understudied.

Therefore, this mini review aims to synthesize the current evidence on neurophysiological and systemic biological effects of music-based interventions in ADHD and to examine how these findings relate to reported behavioral outcomes and potential underlying mechanisms. Given the limited number of studies, substantial methodological heterogeneity, and the lack of standardized outcome measures across the literature, a narrative synthesis approach was adopted with a particular focus on methodological quality, mechanistic interpretation, and translational relevance.

### 1.1 Search strategy and selection criteria

A structured literature search was conducted in PubMed and PsycINFO to identify studies examining music-based interventions, musical activities, music-related paradigms, and their neurophysiological or biological correlates in ADHD. Search terms included ADHD-related terms (e.g., “ADHD”, “attention-deficit hyperactivity disorder”, “attention-deficit/hyperactivity disorder”), music-related terms (e.g., “music”, “music therapy”, “music intervention”, “music listening”, “musical training”, “music making”), and neurophysiological or biological outcome terms (e.g., “EEG”, “MEG”, “MRI”, “auditory processing”, “biomarker”, “cortisol”, “serotonin”, “heart rate variability”). Searches were performed using Boolean operators (AND, OR), and articles published up to May 20, 2026, were considered.

The core database search identified 119 records in PubMed and 150 in PsycINFO. These were screened for relevance by examining titles, abstracts, and the full text where available. Of these, 37 records were identified as directly relevant to the objectives of the present review. Systematic forward citation tracking and reference list screening were not performed. The final synthesis focused on studies meeting all eligibility criteria and providing information on behavioral, neurophysiological, and/or biological outcomes associated with music-based interventions in ADHD.

Studies were included if they (i) involved participants with a formal ADHD diagnosis, (ii) investigated a music-based intervention, musical activity, or music-related experimental paradigm, (iii) reported behavioral, neurophysiological, or biological outcomes, (iv) were published in English, and (v) provided full-text access. Given the limited number of eligible studies and the substantial heterogeneity in intervention modalities, study designs, diagnostic frameworks, and outcome measures, a formal risk-of-bias assessment and quantitative synthesis were beyond the scope of this narrative mini review.

## 2 Effects of musical interventions

### 2.1 Methodological considerations and evidence from music-based interventions

The interpretation of music-based interventions in ADHD is complicated by substantial heterogeneity across the literature. **Table 1** summarizes available studies investigating music-based interventions in ADHD and highlights the considerable variability. Studies differ regarding diagnostic frameworks, ADHD presentations, medication status, intervention modalities, study designs, and outcome measures. This limits comparability across studies and complicates the interpretation of music-related findings.

**Table 1:** Characteristics and main findings of studies on music-based interventions in ADHD focusing on behavioral and clinical outcomes.

Study	Design	N	Population & diagnosis criteria for inclusion	Intervention type	Key findings	Neurophysiological markers and/or systemic biological markers
(23)	Pilot study with wait-list control group	15 (majority diagnosed with ADHD, n=10; rest unclear)	Aggressive adolescent boys (age 11–15 yrs); diagnosed with ADHD according to DSM-IV criteria; additional comorbidities common; approx. 50% medicated with stimulants.	Music therapy (n = 11) versus wait-list control (n = 4). Group music therapy focused on promoting prosocial behavior through structured and semi-structured music activities, group music-making, improvisation, and peer interaction (30–45 min, twice weekly).	Reduced aggression and improved prosocial behavior; interpretation limited by baseline differences in age and diagnostic composition between groups	Not reported
(24)	Comparative study with wait-list control group	13 (all ADHD)	Adolescent boys aged 11–16 years with DSM-IV ADHD in a residential education facility; all receiving stimulant medication; learning difficulties and psychiatric comorbidities reported.	Instructional versus improvisational group music therapy; wait-list control (n = 5), improvisational-first MT (n = 4), and instructional-first MT (n = 4). The instructional approach included structured rhythm-based training, whereas the improvisational approach focused on non-directive group improvisation and emotional expression (8 sessions per condition).	Reduced motor impulsivity; no significant differences between instructional and improvisational music therapy approaches	Not reported
(29)	RCT	120 (all ADHD; n = 40 ADHD-I, n = 33 ADHD-H, n = 47 ADHD-C)	Children aged 2–7 yrs diagnosed with ADHD according to DSM-IV criteria.	Music therapy combined with cognitive-behavioral intervention (16-week intervention; control group received no intervention; n = 60 per group).	Reported improvements in cognitive and behavioral outcomes	Not reported
(25)	RCT	36 (all ADHD)	Children and adolescents (mean age: 12.06 ± 2.63 yrs*); diagnosed with ADHD by a university hospital and presenting with clinically relevant depressive symptoms (CDI > 21); no specific ADHD-manual referenced in methods section.	Music therapy + standard care (12-week intervention; control group received standard care only (n = 18); music therapy (n = 18): active music therapy (improvisation) and receptive music therapy (music listening) 50 min twice a week; total = 24 times).	Reduced depression and stress	Biochemical markers (increased plasma serotonin levels and decreased cortisol levels), reduced heart rate and blood pressure.
(28)	RCT	60 (all ADHD + ODD)	Children (mean age = 4.98 ± 0.77 yrs); diagnosed with comorbid ADHD and ODD by their psychiatrist and confirmed by the school pediatrician (inattentive, hyperactive/impulsive, and combined types included; no specific ADHD-manual referenced in methods section).	Music listening and yoga-based multimodal intervention (16-week intervention; combined yoga + music, yoga-only, music-only and control groups; control group received no intervention; n = 15 per group).	Reduced SNAP-IV scores (reported standardized effect size within-group d = 0.48–0.93)	Not reported
(27)	Pre-post study	13 (all ADHD; n = 2 ADHD-I, n = 11 ADHD-C)	Children (6.09 ± 0.72 yrs) diagnosed with ADHD according to DSM-5 criteria.	Music + movement (8-week intervention; 1 hour per week, singing prominent Taiwanese nursery rhymes and corresponding body movements).	Improved attentional performance (shorter K-CPT 2 hit reaction times), improved quality of life (PedsQL), no significant changes in SNAP-IV	EEG (increased alpha power in the C3 and O1 channels, decreased delta power in the P3 channel, and increased Higuchi’s fractal dimension in the T6 and O2 channels).
(26)	RCT	39 (all ADHD)	Children aged 5–12 yrs with a clinical ADHD diagnosis (no standardized diagnostic interview specified in the methods section; children with prior musical instrument experience were specifically excluded).	Music-based occupational therapy (6-week intervention; weekly 45 min sessions involving harmonica- and drum-based activities [n = 19]; control group received structured occupational therapy [n = 20]).	Improved attention levels, executive functions, and caregiver burden (all maternal caregiver assessment). Significantly greater improvements in attention in music-based occupational therapy group than in structured occupational therapy group.	Not reported
(30)	RCT	64 (all ADHD; n = 21 ADHD-I, n = 15 ADHD-H, n = 28 ADHD-C)	Children aged 6–10 yrs diagnosed with ADHD according to DSM-5 criteria.	Rhythmic music game intervention (8-week intervention; 40 min sessions three times weekly; approx. 960 min total intervention time; n = 32). The control group received passive routine health education (approx 80 min total; n = 32).	Significant improvements in inhibitory control, cognitive flexibility, ADHD symptom severity, and sensorimotor coordination in the intervention group (fewer SCWT errors, shorter TMT-B completion times, lower ARS-5 scores, and improved TGMD-3 scores). Between-group comparisons showed statistically significant effects favoring the intervention group (p < 0.01; several outcomes p < 0.001).	Not reported

*Overall mean age was calculated from individual participant values reported in the publication. Except for Rickson (2006) and Rickson & Watkins (2003), all studies included both boys and girls.

ADHD, Attention-Deficit/Hyperactivity Disorder; ADHD-C, ADHD combined type; ADHD-H, ADHD hyperactive/impulsive type; ADHD-I, ADHD inattentive type; ARS-5, ADHD Rating Scale-5; CDI, Children’s depression inventory; DSM-IV/5, Diagnostic and Statistical Manual of Mental Disorders, Fourth or Fifth Edition; EEG, electroencephalography; K-CPT 2, Conners’ Kiddie Continuous Performance Test; ODD, Oppositional Defiant Disorder; PedsQL, Pediatric Quality of Life Inventory; RCT, randomized controlled trial; SCWT, Stroop Color Word Test; SNAP-IV (Swanson, Nolan, and Pelham Rating Scale; TGMD-3, Test of Gross Motor Development, Third Edition; TMT, Trail Making Test.

In this review, the term “music-based interventions” is used as an umbrella term encompassing therapist-led music therapy, structured music listening, musical training, and multimodal interventions integrating music with additional therapeutic components. These approaches differ considerably in therapeutic intention, delivery format, and the extent to which music represents the primary active intervention component. In clinically diagnosed ADHD populations, only a small number of studies have investigated music-based interventions. Early studies suggested potential improvements in aggression and motor impulsivity following music therapy in adolescents with ADHD (23, 24). However, interpretation is limited by small sample sizes, significant baseline differences in age and diagnostic composition between groups, inconsistent reporting of standardized effect sizes, and insufficient control of medication effects, as many participants received concurrent stimulant treatment.

More recent studies have increasingly examined music within multimodal intervention frameworks. Adjunctive music therapy has been associated with improvements in stress coping and depressive symptoms in children and adolescents with ADHD (25) and produced more pronounced positive effects in terms of caregiver-reported attention levels, executive functions, and caregiver burden compared to occupational therapy activities without music (26). However, findings were based solely on maternal caregiver reports (26). In addition, interventions combining music with movement, gamified rhythmic training, or other multimodal approaches have reported preliminary improvements in attentional and behavioral domains (27–30). Nevertheless, intervention intensity differed between groups. Specifically, Lee et al. (27) reported improvements in attention and quality of life, although ADHD symptom ratings remained unchanged. Zhu (30) found improvements in executive functions, sensorimotor abilities, and ADHD symptoms following rhythmic music game-based interventions. Because music was not evaluated as an isolated intervention component, music-specific effects remain unclear.

Across intervention studies, reported improvements most frequently involve domains related to attention, impulse control, emotional regulation, and broader behavioral functioning. However, the magnitude, specificity, and clinical relevance of these effects remain difficult to determine because of considerable variability in intervention formats, study designs, and outcome assessment (22, 31). While some studies have examined core ADHD-related outcomes such as attention, executive functioning, inhibitory control, and symptom severity (26–30), other studies focused predominantly on associated domains including aggression, depressive symptoms, or stress coping (23, 25). Consequently, the degree to which music-based interventions specifically affect core ADHD symptoms remains incompletely understood.

Meta-analytic evidence remains sparse and highly heterogeneous. Goes et al. (31) synthesized a small number of studies and reported a large but non-significant pooled effect accompanied by extremely high heterogeneity, preventing reliable conclusions regarding efficacy. Taken together, current evidence suggests that music-based interventions may influence behavioral and regulatory domains relevant to ADHD. However, robust conclusions regarding the efficacy of music therapy as an isolated intervention are not yet warranted. Given these methodological limitations and the difficulty of isolating music-specific behavioral effects, neurophysiological and biological measures may provide a complementary mechanistic level of analysis. The following sections summarize current evidence on neurophysiological and systemic biological correlates of music-based interventions in ADHD (see **Table 2**).

**Table 2:** Characteristics and main findings of supporting studies on neurophysiological and/or systemic biological markers in music-based measures in ADHD.

Study	Design	N	Population & diagnosis criteria for inclusion	Intervention type	Key findings	Neurophysiological markers and/or systemic biological markers
(16)	Longitudinal observational cohort study with two measurement time points 13 months apart	132 (n = 111 typically developing, n = 21 ADHD/ADD)	Children aged 7–9 yrs; ADHD/ADD group: diagnosis by pediatrician or child psychologist; ICD-10 F98.8 ADD or F90 ADHD included.	Musical training/instrumental practice exposure, not therapy. Children varied in extracurricular/private lessons and/or the German “JeKi” school instrument program; musical practice quantified with cumulative IMP.	High-practicing children showed larger, especially right-hemispheric HG volumes, and stronger bilateral P1 synchronization. ADHD/ADD children showed smaller HG, enlarged PT, lower HG/PT ratios, and pronounced bilateral P1 asynchrony.	Structural MRI: gray matter volumes of HG and PT; HG/PT ratio. MEG: auditory-evoked P1 amplitude, P1 latency, right–left P1 asynchrony.
(32)	Mainly cross-sectional MRI/MEG/psychoacoustic study; additional longitudinal sub-sample over 3.6 years examining musical practice	147 (n = 37 control, n = 37 dyslexia, n = 37 ADHD, n = 36 ADD; plus n = 15 comorbid cases shown separately. Longitudinal sub-sample: n = 109)	Children around 10–11 yrs. ADHD/ADD diagnoses made by child psychiatrists, psychologists, or pediatricians. ADHD included ICD-10 F90.0/F90.1; ADD included ICD-10 F98.8. IQ <80 and known comorbidities such as dyscalculia, autism, and epilepsy were excluded from the main study.	Musical training/instrumental practice exposure, not therapy; classified by IMP. In the longitudinal analysis, children were classified as musicians if IMP >4 and non-musicians if IMP ≤4.	ADHD and ADD showed smaller left HG, enlarged left PT, lower left HG/PT ratios, and pronounced bilateral P1 asynchrony compared with controls. ADHD showed additional right-sided abnormalities, including lower right HG/PT ratios and diminished right P1 amplitudes, whereas ADD showed preserved right HG morphology and more balanced, time-shifted bilateral P1 waveforms. Musical training was associated with shorter, more synchronized P1 responses, especially in the pooled disorder group.	Structural MRI: gray matter volumes of HG and PT; HG/PT ratio. MEG: auditory-evoked P1 latency, P1 amplitude, P1 width, absolute right–left P1 asynchrony, asymmetry indices ΔL, ΔW, ΔA.
(41)	Controlled trial (pilot + phase 2)	70 (n = 20 pilot [n = 10 ADHD, n = 10 typically developing] and n = 50 phase 2 [n = 25 ADHD, n = 25 typically developing])	Preadolescents (mean age = 12.05 ± 1.18 yrs [pilot] and 10.36 ± 1.48 yrs [phase 2*]); diagnosis provided by expert medical professionals with specialized training and experience in ADHD diagnostics; no specific manual referenced.	Exposure to (i) no music, (ii) calm music no lyrics, (iii) calm music with lyrics, and (iv) rhythmic music with lyrics (ordering of music conditions was identical for all participants).	Increased reading comprehension scores with background music in ADHD groups but not typically developed group; convergence in performance between the two groups in music-conditions.	Reduced decreases in HRV; significant in calm music with and w/o lyrics; reduced but not significant in rhythmic music.
(42)	Retrospective study of clinical data	122 (all tic disorders; n = 46 comorbid ADHD)	Children with tic disorders (age 5–14 yrs); no specific manual referenced for ADHD diagnosis.	Music therapy + habit reversal training (8-week intervention, combined [n = 67 total, n = 23 (34%) comorbid ADHD] or HRT-only [n = 55 total, n = 23 (41%) comorbid ADHD]).	Significant decrease in tic symptoms, significant improvements in behavior and quality of life (stronger effects in combined-group than HRT-only, p < 0.05).	Biochemical (decreased serum levels of dopamine and serotonin, increased levels of γ-aminobutyric acid; all combined-group significantly stronger effects than HRT-only; p < 0.05).

*Overall mean age was calculated from individual participant values reported in the publication. All studies included both boys and girls.

ADD, attention deficit disorder; ADHD/ADD, attention-deficit (hyperactivity) disorder, including ADHD and ADD); ADHD, Attention-Deficit/Hyperactivity Disorder; DSM-IV, Diagnostic and Statistical Manual of Mental Disorders, Fourth Edition; HG, Heschl’s gyrus; HRT, habit reversal training; HRV, heart-rate variability; ICD-10, International Statistical Classification of Diseases and Related Health Problems, 10th Revision; IMP, index of cumulative musical practice; MEG, magnetoencephalography; MRI, magnetic resonance imaging; PT, planum temporale.

### 2.2 Neurophysiological effects of musical interventions in ADHD

The literature on neurophysiological effects of music in ADHD remains limited and heterogeneous. In children, Seither-Preisler et al. (16) showed that musical practice was associated with structural and functional characteristics of the auditory cortex. Using structural MRI and MEG, children with ADHD/ADD (diagnosed according to ICD-10; see **Table 2**) had reduced Heschl’s gyrus (HG) and enlarged planum temporale (PT) volumes, particularly in the left hemisphere, resulting in a lower HG/PT ratio. Functionally, they showed atypical lateralization with accelerated right-hemispheric and delayed left-hemispheric responses, leading to increased interhemispheric P1 asynchrony compared with musically experienced typically developing children. Smaller left HG/PT ratios and greater bilateral P1 asynchrony were associated with stronger ADHD/ADD symptoms. By contrast, musical practice was associated with larger HG volumes, larger and faster auditory responses, and greater interhemispheric synchronization. Longitudinally, auditory cortex morphology remained stable and MEG patterns were reproducible, but P1 latency decreased by about 5 ms over 13 months, consistent with normal maturation; this reduction was smallest in children with ADHD/ADD and largest in highly musically active typically developing children, suggesting delayed auditory maturation in ADHD/ADD and accelerated maturation with musical experience (16).

Serrallach et al. (32) examined auditory-cortex biomarkers in children with dyslexia, ADHD, and ADD using MRI, MEG, and psychoacoustics. Both ADHD and ADD (diagnosis according to ICD-10) showed atypical left auditory-cortex morphology, including smaller left HG, enlarged left PT, reduced HG/PT ratios, and increased interhemispheric P1 asynchrony. In controls, P1 sources localized to left and right HG, whereas all disorder subgroups showed a more posterior P1 source in the PT. Controls exhibited well-balanced bilateral responses with small asynchrony, while P1 asynchrony was about five times greater in all disorder groups. Importantly, ADHD and ADD were not neurophysiologically identical: children with ADHD showed reduced right HG/PT ratios, diminished and sharpened right-hemispheric P1 responses, temporally expanded responses in the left hemisphere, and poorer rhythm and melody processing, whereas children with ADD showed a more normal-sized right HG, similar but time-shifted bilateral waveform shapes, and no clear auditory impairments. In a longitudinal subsample followed over 3.6 years, musical training did not alter gross auditory-cortex morphology but was associated with shorter more synchronized auditory responses; children with higher cumulative musical practice showed lower absolute P1 asynchrony, a mean reduction of 5.5 ± 1.0 ms, and stronger synchronization effects in the pooled disorder group than in controls (32). These findings suggest a potential association between musical engagement and improved interhemispheric synchronization of auditory processing in developmental disorders, including ADHD.

Lee et al. (27) provide EEG evidence, reporting resting-state changes after an 8-week music-and-movement intervention in children with ADHD (combined and inattentive presentations according to DSM-5; see **Table 1**). Alpha power increased in C3/O1, delta power decreased in P3, and Higuchi’s fractal dimension increased in T6/O2, suggesting altered cortical oscillatory activity and signal complexity. This study provides preliminary evidence that music-and-movement therapy may modulate neural activity in ADHD, although evidence is limited to a short pre–post design.

Overall, music-related interventions may affect brain function in ADHD at several levels, including auditory-cortex morphology–function relationships, interhemispheric timing and synchronization, and resting-state EEG oscillations. The evidence base remains small, diagnostically heterogeneous, and methodologically diverse, so conclusions are preliminary, and the extent to which these neurophysiological changes translate into clinically meaningful behavioral improvements remains unclear.

### 2.3 Systemic biological effects of music interventions

Although ADHD is primarily characterized by its behavioral symptoms, analyses of systemic consequences based on blood, saliva, and urine samples have identified alterations in selected biochemical markers (e.g., hormonal, immunological, and metabolic systems - reviewed in (33–36)). Collectively, individuals with ADHD exhibit systemically measurable alterations in stress-related, inflammatory, and neuroendocrine pathways, including significant increases in interleukin-6 (IL-6) and reductions in tumor necrosis factor alpha (TNF-α), oxidative and nitrosative stress, and altered cortisol levels, a key mediator of the hypothalamic–pituitary–adrenal (HPA) axis (35, 37–39). A comprehensive view of ADHD’s biochemical implications remains elusive.

Biochemical responses to treatment have primarily been studied in pharmacological interventions. For instance, methylphenidate treatment has been associated with improved physiological stress markers in children and adults with ADHD, including reductions in oxidative and/or nitrosative stress as well as changed cortisol levels (39, 40).

In comparison, data on the systemic biological effects of non-pharmacological interventions are scarce, with only a few studies reporting biochemical or physiological markers following music-based interventions in individuals with ADHD. Park et al. (25) were the only ones to include an assessment of biochemical markers (see **Table 1**). There, children with ADHD receiving music therapy exhibited significantly increased plasma serotonin levels alongside reduced cortisol concentrations, whereas no such changes were observed in the non-intervention group. Music therapy was additionally associated with reductions in heart rate and blood pressure, suggesting a modulatory effect on autonomic and stress-related systems. This observation corroborated previous findings indicating that exposure to music can attenuate decreases in heart-rate variability (HRV; **Table 2**), an established proxy of autonomic nervous system regulation, in children with ADHD but not in typically developed children (41).

Given their considerable rate of ADHD-comorbidities (34–41%), the retrospective study in children with tic disorders, in which music therapy was combined with habit reversal training (HRT), provides additional insights into potential systemic effects (42). There, both groups showed decreased serum dopamine and serotonin and increased γ-aminobutyric acid (GABA) levels. The combination of music therapy and HRT showed significantly stronger effects than the HRT-only group, suggesting an enhanced modulatory effect in the music therapy group.

Despite these promising observations and the clear need for a better understanding of the systemic effects of therapeutic interventions such as music therapy, the current evidence base remains too limited to draw reliable conclusions. Moreover, methodological limitations (e.g., insufficient consideration of diurnal marker variations, lack of correction for multiple comparisons) and limited integration of multi-omics approaches or longitudinal study designs, further limit mechanistic interpretations.

Taken together, neurophysiological and systemic findings point toward a multi-level modulation of processes relevant to ADHD, including auditory processing, neural synchronization, and stress-related regulatory systems. However, the lack of studies concurrently integrating behavioral, neural, and biological outcomes represents a major limitation, warranting further investigations.

## 3 Discussion

### 3.1 Clinical relevance, transdiagnostic context and ADHD-specific rationale

Music-based interventions are increasingly discussed as complementary approaches, as health care systems need accessible, effective, and preventive interventions beyond symptom management alone. Pharmacological and behavioral treatments remain central in many psychiatric and neurodevelopmental conditions, including ADHD (17), but unmet clinical needs remain, particularly for individuals who experience partial benefit, variable responses, or limited tolerability. In this context, music therapy could represent a promising therapeutic approach because it combines emotional engagement, social interaction, attentional demands, temporal structure, and sensorimotor integration within a potentially motivating and individualized intervention format (43–45).

Recent transdiagnostic evidence suggests that music-based interventions may improve psychiatric symptoms, quality of life, and subjective well-being across a range of clinical and non-clinical populations (46, 47). These findings support the broader clinical relevance of music-based approaches beyond ADHD but may be particularly relevant for ADHD because musical activities engage attentional, executive, temporal, sensorimotor, and emotional regulation (4, 5, 48) that overlap substantially with affected domains. Rhythm, auditory-motor coupling, and temporal prediction may represent plausible mechanisms linking musical engagement to ADHD-related symptoms. Preliminary neurophysiological findings further support this conceptual link by suggesting associations with auditory processing, and neural synchronization (16, 27, 32).

### 3.2 Current ADHD-specific evidence

Despite growing interest in music-based approaches for ADHD, the current evidence base remains limited. Only a small number of studies have investigated music-based interventions in clinically diagnosed ADHD populations, with substantial variability in intervention formats, outcome measures, diagnostic approaches, and study designs (**Table 1**).

Early studies suggested potential improvements in behavioral and regulatory domains in adolescents with ADHD (23, 24), while more recent studies have examined music within multimodal intervention frameworks, reporting preliminary improvements in attentional, emotional, and behavioral domains (25–30). However, because music was rarely evaluated as an isolated intervention component, the specific contribution of music to the observed effects remains difficult to determine.

Across studies, reported improvements most frequently involve attention, impulse control, emotional regulation, and broader behavioral functioning. However, many studies also focused on associated domains (e.g., aggression, stress coping, or depressive symptoms) rather than core ADHD symptoms (23, 25, 29), relied heavily on caregiver assessments (26), and rarely reported null effects.

Meta-analytic evidence remains sparse (31). Despite the limitations discussed above, current findings suggest potential benefits of music-based interventions for behavioral and regulatory domains relevant to ADHD, although conclusions regarding efficacy and mechanistic specificity remain preliminary.

### 3.3 Neurophysiological and biological mechanisms

Beyond behavioral outcomes, neurophysiological and systemic biological markers represent a crucial but underdeveloped field. Evidence from broader music neuroscience research suggests that musical interventions may influence auditory, attentional, neuroendocrine, autonomic, and stress-related regulatory processes (19, 21, 49).

However, within ADHD, findings remain particularly sparse: Park et al. currently represents the only ADHD-specific music-based intervention study to include systemic outcomes, reporting changes in peripheral biochemical markers, heart rate, and blood pressure (25). Corroborated by HRV-decreases (41), this indicates modulatory effects on stress-related and autonomic regulatory systems.

Similarly, neurophysiological evidence from studies using MRI, MEG, and EEG suggests that musical interventions and musical engagement may affect auditory cortex function, timing, interhemispheric synchronization, and cortical oscillations (16, 27, 32). These domains are highly relevant to ADHD given the established roles of timing deficits, auditory-attentional integration, and executive dysfunction. However, the available evidence remains preliminary and is limited by diagnostic heterogeneity, methodological variability, and insufficient biological contextualization.

### 3.4 Diagnostic and methodological heterogeneity

The interpretation of these findings is complicated not only by the heterogeneity of ADHD itself, but by inconsistent diagnostic approaches across individual studies and classification systems. Differences between DSM-IV, DSM-5, ICD-10, and ICD-11 classification systems further complicate comparability across studies, particularly regarding inattentive, hyperactive-impulsive, and combined ADHD presentations and whether they include ADHD and/or ADD, which is directly relevant for music-related ADHD research. This is important because music-based interventions may not affect all ADHD domains equally, rendering a direct relevance for music-related ADHD research; for example, rhythm- and movement-based approaches could be more relevant for motor impulsivity and temporal processing, whereas receptive music listening or structured auditory stimulation could potentially affect sustained attention or emotional regulation. Thus, potentially meaningful differences between inattentive, hyperactive-impulsive, and combined presentations remain largely unexplored.

### 3.5 Future research directions

Here we report that neither neurophysiological nor systemic biological measures are typically integrated with behavioral outcomes within the same study; their concurrent inclusion remains particularly rare. Future research on neurophysiological, biochemical and systemic measures is therefore required and should include larger, adequately powered samples, standardized ADHD diagnostic criteria and sampling protocols, and careful considerations of relevant confounding factors (e.g., medication status, core ADHD presentations/comorbidities, diurnal marker variation or developmental stage). Moreover, future studies should consider that biological measures are not generic “stress markers” but embedded in complex regulatory frameworks (50).

Finally, ADHD-specific evaluation is hampered by the limited developmental scope represented in the current literature. Although ADHD is increasingly recognized as a lifespan condition (3) all intervention studies included in this review focused exclusively on children or adolescents, and evidence in adults with ADHD remains largely absent. Importantly, this gap represents not only a limitation of the current evidence base but also a relevant neurobiological research question. Findings obtained in developing brains may not directly generalize to adulthood because neurodevelopmental trajectories, neural plasticity mechanisms, and network maturation differ substantially across the lifespan. In addition, compensatory cognitive and behavioral strategies often become increasingly consolidated with age, potentially altering both baseline neurophysiological functioning and responsiveness to interventions. Adult ADHD is also characterized by distinct patterns of psychiatric and somatic comorbidity, which may further influence biological and behavioral treatment responses (3). Consequently, future studies should explicitly investigate adult ADHD populations and adopt longitudinal designs to determine whether music-related neurophysiological and biological effects differ across developmental stages, which markers predict clinical response, and which intervention components contribute to therapeutic effects across the lifespan.

## 4 Conclusion

In conclusion, music-based interventions offer a promising but insufficiently characterized approach for ADHD. Their relevance is supported by broader psychiatric evidence, by the conceptual overlap between musical processes and ADHD-related domains, and by preliminary findings suggesting behavioral, neurophysiological, and systemic biological modulation. Nevertheless, the current evidence base is limited by heterogeneity, small sample sizes, incomplete reporting, insufficient mechanistic integration, and a marked lack of adult data. A more standardized, biologically informed, and multimodal research agenda is needed to determine whether and how music therapy can contribute to individualized and mechanism-based care for ADHD.

## References

[1] Polanczyk G de Lima MS Horta BL Biederman J Rohde LA . The worldwide prevalence of ADHD: a systematic review and metaregression analysis. Am J Psychiatry. (2007) 164:942–8. doi: 10.1176/ajp.2007.164.6.942 17541055

[2] Song P Zha M Yang Q Zhang Y Li X Rudan I . The prevalence of adult attention-deficit hyperactivity disorder: a global systematic review and meta-analysis. J Glob Health. (2021) 11:4009. doi: 10.7189/jogh.11.04009 33692893 PMC7916320

[3] Cortese S Bellgrove MA Brikell I Franke B Goodman DW Hartman CA . Attention-deficit/hyperactivity disorder (ADHD) in adults: evidence base, uncertainties and controversies. World Psychiatry. (2025) 24:347–71. doi: 10.1002/wps.21374 40948064 PMC12434367

[4] Barkley RA Edwards G Laneri M Fletcher K Metevia L . Executive functioning, temporal discounting, and sense of time in adolescents with attention deficit hyperactivity disorder (ADHD) and oppositional defiant disorder (ODD). J Abnorm Child Psychol. (2001) 29:541–56. doi: 10.1023/a:1012233310098 11761287

[5] Noreika V Falter CM Rubia K . Timing deficits in attention-deficit/hyperactivity disorder (ADHD): evidence from neurocognitive and neuroimaging studies. Neuropsychologia. (2013) 51:235–66. doi: 10.1016/j.neuropsychologia.2012.09.036 23022430

[6] Smith A Taylor E Rogers JW Newman S Rubia K . Evidence for a pure time perception deficit in children with ADHD. J Child Psychol Psychiatry. (2002) 43:529–42. doi: 10.1111/1469-7610.00043 12030598

[7] Volkow ND Wang GJ Kollins SH Wigal TL Newcorn JH Telang F . Evaluating dopamine reward pathway in ADHD: clinical implications. JAMA. (2009) 302:1084–91. doi: 10.1001/jama.2009.1308 19738093 PMC2958516

[8] Del Campo N Chamberlain SR Sahakian BJ Robbins TW . The roles of dopamine and noradrenaline in the pathophysiology and treatment of attention-deficit/hyperactivity disorder. Biol Psychiatry. (2011) 69:e145–57. doi: 10.1016/j.biopsych.2011.02.036 21550021

[9] Alsoqih NS Elmorsy EA Abu-Khudir R Saber S Keshk N Elnaghy F . A systems biology perspective on childhood ADHD: neurochemical dysregulation, brain-behavior interactions, and emerging therapeutics. FASEB J. (2025) 39:e70981. doi: 10.1096/fj.202501829RR 40905754

[10] Banerjee E Nandagopal K . Does serotonin deficit mediate susceptibility to ADHD? Neurochem Int. (2015) 82:52–68. doi: 10.1016/j.neuint.2015.02.001 25684070

[11] Salimpoor VN Benovoy M Larcher K Dagher A Zatorre RJ . Anatomically distinct dopamine release during anticipation and experience of peak emotion to music. Nat Neurosci. (2011) 14:257–62. doi: 10.1038/nn.2726 21217764

[12] Ferreri L Mas-Herrero E Cardona G Zatorre RJ Antonijoan RM Valle M . Dopamine modulations of reward-driven music memory consolidation. Ann N Y Acad Sci. (2021) 1502:85–98. doi: 10.1111/nyas.14656 34247392

[13] Dawes P Bishop D . Auditory processing disorder in relation to developmental disorders of language, communication and attention: a review and critique. Int J Lang Commun Disord. (2009) 44:440–65. doi: 10.1080/13682820902929073 19925352

[14] Riccio CA Cohen MJ Garrison T Smith B . Auditory processing measures: correlation with neuropsychological measures of attention, memory, and behavior. Child Neuropsychol. (2005) 11:363–72. doi: 10.1080/09297040490916956 16051564

[15] Scheich H Brechmann A Brosch M Budinger E Ohl FW Selezneva E . Behavioral semantics of learning and crossmodal processing in auditory cortex: the semantic processor concept. Hear Res. (2011) 271:3–15. doi: 10.1016/j.heares.2010.10.006 20971178

[16] Seither-Preisler A Parncutt R Schneider P . Size and synchronization of auditory cortex promotes musical, literacy, and attentional skills in children. J Neurosci. (2014) 34:10937–49. doi: 10.1523/JNEUROSCI.5315-13.2014 25122894 PMC6705250

[17] Cortese S Adamo N Del Giovane C Mohr-Jensen C Hayes AJ Carucci S . Comparative efficacy and tolerability of medications for attention-deficit hyperactivity disorder in children, adolescents, and adults: a systematic review and network meta-analysis. Lancet Psychiatry. (2018) 5:727–38. doi: 10.1016/S2215-0366(18)30269-4 30097390 PMC6109107

[18] Luo Y Weibman D Halperin JM Li X . A review of heterogeneity in attention deficit/hyperactivity disorder (ADHD). Front Hum Neurosci. (2019) 13:42. doi: 10.3389/fnhum.2019.00042 30804772 PMC6378275

[19] Chanda ML Levitin DJ . The neurochemistry of music. Trends Cognit Sci. (2013) 17:179–93. doi: 10.1016/j.tics.2013.02.007 23541122

[20] Thaut MH Hoemberg V . Handbook of Neurologic Music Therapy. 2. ed. New York: Oxford University Press (2024).

[21] Koelsch S . Brain correlates of music-evoked emotions. Nat Rev Neurosci. (2014) 15:170–80. doi: 10.1038/nrn3666 24552785

[22] Saville P Kinney C Heiderscheit A Himmerich H . Exploring the intersection of ADHD and music: a systematic review. Behav Sci (Basel). (2025) 15(1):65. doi: 10.3390/bs15010065 39851869 PMC11762814

[23] Rickson DJ Watkins WG . Music therapy to promote prosocial behaviors in aggressive adolescent boys--a pilot study. J Music Ther. (2003) 40:283–301. doi: 10.1093/jmt/40.4.283 15015908

[24] Rickson DJ . Instructional and improvisational models of music therapy with adolescents who have attention deficit hyperactivity disorder (ADHD): a comparison of the effects on motor impulsivity. J Music Ther. (2006) 43:39–62. doi: 10.1093/jmt/43.1.39 16671837

[25] Park JI Lee IH Lee SJ Kwon RW Choo EA Nam HW . Effects of music therapy as an alternative treatment on depression in children and adolescents with ADHD by activating serotonin and improving stress coping ability. BMC Complement Med Ther. (2023) 23:73. doi: 10.1186/s12906-022-03832-6 36879223 PMC9987133

[26] Erarslan I Budak M Tarakci D . Effects of music-based occupational therapy activities on attention executive functions in children with attention deficit and hyperactivity disorder. PloS One. (2026) 21:e0349284. doi: 10.1371/journal.pone.0349284 42149817 PMC13183191

[27] Lee MW Yang NJ Mok HK Yang RC Chiu YH Lin LC . Music and movement therapy improves quality of life and attention and associated electroencephalogram changes in patients with attention-deficit/hyperactivity disorder. Pediatr Neonatol. (2024) 65:581–7. doi: 10.1016/j.pedneo.2023.11.007 38641441

[28] Luo X Huang X Lin S . Yoga and music intervention reduces inattention, hyperactivity/impulsivity, and oppositional defiant disorder in children's consumer with comorbid ADHD and ODD. Front Psychol. (2023) 14:1150018. doi: 10.3389/fpsyg.2023.1150018 37809284 PMC10552923

[29] Zhu C . Effects of musicotherapy combined with cognitive behavioral intervention on the cognitive ability of children with attention deficit hyperactivity disorder. Psychiatr Danub. (2022) 34:288–95. doi: 10.24869/psyd.2022.288 35772139

[30] Zhu H . Influence of rhythmic music game intervention on executive function and sensorimotor ability of children with attention deficit hyperactivity disorder. Front Public Health. (2026) 14:1808386. doi: 10.3389/fpubh.2026.1808386 42145502 PMC13176269

[31] Goes AO Nardi AE Quagliato LA . Outcomes of music therapy on children and adolescents with attention-deficit/hyperactivity disorder: a systematic review and meta-analysis. Trends Psychiatry Psychother. (2025) 47:e20241020. doi: 10.47626/2237-6089-2024-1020 40680190 PMC12962369

[32] Serrallach B Gross C Bernhofs V Engelmann D Benner J Gundert N . Neural biomarkers for dyslexia, ADHD, and ADD in the auditory cortex of children. Front Neurosci. (2016) 10:324. doi: 10.3389/fnins.2016.00324 27471442 PMC4945653

[33] Bonvicini C Faraone SV Scassellati C . Attention-deficit hyperactivity disorder in adults: a systematic review and meta-analysis of genetic, pharmacogenetic and biochemical studies. Mol Psychiatry. (2016) 21:872–84. doi: 10.1038/mp.2016.74 27217152 PMC5414093

[34] Fulun L Yanan L Bing G Qian L Aishu L Xia L . Hypothalamic-pituitary-adrenal axis dysfunction in children with ADHD: a systematic review and meta-analysis. Psychoneuroendocrinology. (2025) 181:107605. doi: 10.1016/j.psyneuen.2025.107605 40974807

[35] Misiak B Wojta-Kempa M Samochowiec J Schiweck C Aichholzer M Reif A . Peripheral blood inflammatory markers in patients with attention deficit/hyperactivity disorder (ADHD): a systematic review and meta-analysis. Prog Neuro-Psychopharmacol Biol Psychiatry. (2022) 118:110581. doi: 10.1016/j.pnpbp.2022.110581 35660454

[36] Predescu E Vaidean T Rapciuc AM Sipos R . Metabolomic markers in attention-deficit/hyperactivity disorder (ADHD) among children and adolescents-a systematic review. Int J Mol Sci. (2024) 25:4385. doi: 10.3390/ijms25084385 38673970 PMC11050195

[37] Avcil S Uysal P Yenisey C Abas BI . Elevated melatonin levels in children with attention deficit hyperactivity disorder: relationship to oxidative and nitrosative stress. J Atten Disord. (2021) 25:693–703. doi: 10.1177/1087054719829816 30819002

[38] Corona JC . Role of oxidative stress and neuroinflammation in attention-deficit/hyperactivity disorder. Antioxidants (Basel). (2020) 9(11):1039. doi: 10.3390/antiox9111039 33114154 PMC7690797

[39] Garre-Morata L de Haro T Villen RG Fernandez-Lopez ML Escames G Molina-Carballo A . Changes in cortisol and in oxidative/nitrosative stress indicators after ADHD treatment. Antioxidants (Basel). (2024) 13(1):92. doi: 10.3390/antiox13010092 38247516 PMC10812591

[40] Sinningen K Emons B Bohme P Juckel G Hanusch B Beckmann B . l-Arginine/nitric oxide pathway and oxidative stress in adults with ADHD: effects of methylphenidate treatment. Nitric Oxide. (2023) 138-139:64–9. doi: 10.1016/j.niox.2023.06.006 37392928

[41] Madjar N Gazoli R Manor I Shoval G . Contrasting effects of music on reading comprehension in preadolescents with and without ADHD. Psychiatry Res. (2020) 291:113207. doi: 10.1016/j.psychres.2020.113207 32559672

[42] Liu Y Chang H Li X Zhang M Wang X . Effects of music therapy combined with habit reversal training on children with tic disorders: a retrospective study. Noise Health. (2025) 27:104–11. doi: 10.4103/nah.nah_195_24 40298049 PMC12063948

[43] de Witte M Pinho ADS Stams GJ Moonen X Bos AER van Hooren S . Music therapy for stress reduction: a systematic review and meta-analysis. Health Psychol Rev. (2022) 16:134–59. doi: 10.1080/17437199.2020.1846580 33176590

[44] Koelsch S . A neuroscientific perspective on music therapy. Ann N Y Acad Sci. (2009) 1169:374–84. doi: 10.1111/j.1749-6632.2009.04592.x 19673812

[45] Särkämö T Sihvonen AJ . Golden oldies and silver brains: deficits, preservation, learning, and rehabilitation effects of music in ageing-related neurological disorders. Cortex. (2018) 109:104–23. doi: 10.1016/j.cortex.2018.08.034 30312779

[46] Lassner A Siafis S Wiese E Leucht S Metzner S Wagner E . Evidence for music therapy and music medicine in psychiatry: transdiagnostic meta-review of meta-analyses. BJPsych Open. (2024) 11:e4. doi: 10.1192/bjo.2024.826 39668615 PMC11733488

[47] Zhang J Lu Y Mehdinezhadnouri K Liu J Lu H . Impact of music-based interventions on subjective well-being: a meta-analysis of listening, training, and therapy in clinical and nonclinical populations. Front Psychol. (2025) 16:1608508. doi: 10.3389/fpsyg.2025.1608508 40703746 PMC12285531

[48] Shaw P Stringaris A Nigg J Leibenluft E . Emotion dysregulation in attention deficit hyperactivity disorder. Am J Psychiatry. (2014) 171:276–93. doi: 10.1176/appi.ajp.2013.13070966 24480998 PMC4282137

[49] Chee ZJ Chang CYM Cheong JY Malek F Hussain S de Vries M . The effects of music and auditory stimulation on autonomic arousal, cognition and attention: a systematic review. Int J Psychophysiol. (2024) 199:112328. doi: 10.1016/j.ijpsycho.2024.112328 38458383

[50] Fancourt D Ockelford A Belai A . The psychoneuroimmunological effects of music: a systematic review and a new model. Brain Behav Immun. (2014) 36:15–26. doi: 10.1016/j.bbi.2013.10.014 24157429

